# Implementation of a rapid learning platform: Predicting 2-year survival in laryngeal carcinoma patients in a clinical setting

**DOI:** 10.18632/oncotarget.8755

**Published:** 2016-04-15

**Authors:** Tim Lustberg, Michael Bailey, David I. Thwaites, Alexis Miller, Martin Carolan, Lois Holloway, Emmanuel Rios Velazquez, Frank Hoebers, Andre Dekker

**Affiliations:** ^1^ Department of Radiation Oncology (MAASTRO), GROW School for Oncology and Developmental Biology, Maastricht University Medical Centre+, Maastricht, The Netherlands; ^2^ Illawarra Cancer Care Centre, Illawarra Shoalhaven Local Health District, Wollongong, Australia; ^3^ Centre for Oncology Informatics, University of Wollongong, Wollongong, Australia; ^4^ Institute of Medical Physics, School of Physics, University of Sydney, Sydney, Australia; ^5^ Illawarra Health and Medical Research Institute, Wollongong, Australia; ^6^ Centre for Medical Radiation Physics, University of Wollongong, Wollongong, Australia; ^7^ South Western Clinical School, University of New South Wales, Sydney, Australia; ^8^ Ingham Institute and Liverpool and Macarthur Cancer Therapy Centres, Liverpool, Australia; ^9^ Dana-Farber Cancer Institute, Harvard Medical School, Boston, USA

**Keywords:** larynx, survival prediction, rapid learning, model validation

## Abstract

**Background and Purpose:**

To improve quality and personalization of oncology health care, decision aid tools are needed to advise physicians and patients. The aim of this work is to demonstrate the clinical relevance of a survival prediction model as a first step to multi institutional rapid learning and compare this to a clinical trial dataset.

**Materials and Methods:**

Data extraction and mining tools were used to collect uncurated input parameters from Illawarra Cancer Care Centre's (clinical cohort) oncology information system. Prognosis categories previously established from the Maastricht Radiation Oncology (training cohort) dataset, were applied to the clinical cohort and the radiotherapy only arm of the RTOG-9111 (trial cohort).

**Results:**

Data mining identified 125 laryngeal carcinoma patients, ending up with 52 patients in the clinical cohort who were eligible to be evaluated by the model to predict 2-year survival and 177 for the trial cohort. The model was able to classify patients and predict survival in the clinical cohort, but for the trial cohort it failed to do so.

**Conclusions:**

The technical infrastructure and model is able to support the prognosis prediction of laryngeal carcinoma patients in a clinical cohort. The model does not perform well for the highly selective patient population in the trial cohort.

## INTRODUCTION

Laryngeal carcinoma has a recorded incidence of 606 (2009) with 255 reported mortalities (2010) in Australia [1]. In Europe the recorded incidence (2012) was 39,900 [2] and the USA reported an incidence of 12,630 (2014) with an estimated number of deaths of 3,610 [3]. Treatment options for patients with early localized laryngeal carcinoma include surgery or radiotherapy, having equal outcome [4, 5]. For advanced laryngeal cancer, surgery therapy using total laryngectomy has been the standard of care for decades [6], however nowadays laryngeal preservation strategies using primary radiation or chemoradiation have been adopted [7, 8].

New developments to further improve outcome in patients treated with radiotherapy, include the application of dose-escalation [9] and the use of more advanced radiotherapy technologies such as IMRT [10] and proton irradiation [11, 12], to reduce side effects while maintaining local control.

Clinical Decision Support Systems (DSS) are a way to support the choice between the increasing number of radiotherapy techniques and technology options [13–15] both in terms of clinical benefit in the individual patient and in assigning resources to patient groups that benefit most from the new technology to address the concerns of keeping cancer care affordable [16]. To construct a DSS, predictive models need to be learned and validated.

Rapid learning health care is a way to learn predictive models. In rapid learning it is postulated that data routinely generated through patient care and clinical research feed into an ever-growing set of coordinated databases [17]. These coordinated datasets could then be used to learn and validate the model. In this study we present a first rapid learning approach that combines learning a predictive model from one clinical center (“training cohort”) and validating it in both another clinical center (“clinical cohort”) and a clinical trial dataset (“trial cohort”). A comprehensive technical infrastructure is proposed in which databases were coordinated spanning institutions and continents (Maastricht Radiation Oncology (MAASTRO) in Europe, the Radiation Therapy Oncology Group (RTOG) in North-America and Illawarra Cancer Care Centre (ICCC) in Australia).

The hypothesis of this study is that it is possible to implement an automated data extraction infrastructure for rapid learning that uses a model to predict survival in laryngeal carcinoma without any human evaluation of the data to show that routine clinical data is a valuable source of information that can be used to complement the current evidence base consisting mainly of clinical trial data. The model was learned in one institution (MAASTRO) and applied in a patient care-driven regional cancer service (ICCC) and evaluated for a research-driven clinical trial collaborative group (RTOG).

## MATERIALS AND METHODS

### Clinical cohort

After internal review board approval, the data of laryngeal carcinoma patients was extracted from the Oncology Information System (OIS) of ICCC (MOSAIQ, Elekta, Stockholm, Sweden) using a data integration tool (Kettle, Pentaho Community Edition 5.0, Orlando, USA). To provide an automated infrastructure all model input parameters needed to be extracted from the OIS and stored in a data warehouse (MSSQL 2008). Patients were selected using the International Classification of Diseases (ICD) codes version 10. The ICD code for laryngeal carcinoma patients is C32 and all subcategories in this classification group. Patients treated with radiotherapy alone for a primary H&N disease were added to the clinical cohort data warehouse. These patients were diagnosed between April 1987 and February 2014. Over time more patients will be included because it is an automated system in which new patients can be included each time the software is executed. For patients with missing record and verify (R&V) data within the OIS (e.g. due to H&N diagnoses that were manually added to the treatment history for treatment they received elsewhere) an imputation algorithm was added to the data mining script: patients with a H&N diagnosis treated with radiotherapy before 2012 with no recorded delivered dose were assumed to be treated with the recorded prescribed dose. Each of the model parameters were extracted from the OIS with individual data integration programs. Quality assurance of the extracted data was undertaken *via* cross referencing with the OIS.

### H&N predictive model

The data warehouse was queried and analyzed by a predictive model developed using Matlab 8.2.0 (The MathWorks Inc., Natick, MA, USA). The software applies the laryngeal carcinoma survival model [18] to the extracted data and reports the accuracy of the predictions created by the model. The model was fitted with Univariate Cox regression [18] which uses the following factors: age at the time of diagnosis, gender, T-stage, N-stage, hemoglobin level before treatment, tumor location and the biological equivalent dose in fractions of 2 Gray. These features and other features were selected by a medical specialist to be analyzed by the Univariate Cox regression. In the original study [18] it was concluded that these features had a statistical relevance when predicting survival while others had not (e.g. the Tumor Volume computed from the PET scan). This resulted in a model with a baseline two-year survival of 0.1404. Table 1 shows the beta-coefficients of the model and the data input formatting that is used. The proportional hazards model resulting from this fit was implemented as a nomogram in the original study [18] to create an easy to use DSS for the physicians. For this study we implemented the original proportional hazard model as we are using a completely automated digital infrastructure. To evaluate the accuracy of the model the survival in months and the patient deceased status were also extracted from the OIS. To determine the survival of alive patients the last known registered contact within the OIS was used.

**Table 1:** Model coefficients together with the corresponding features and data format

Model Feature	Model Input	Model Beta
Age	number	0.0454
Gender is male?	0/1	0.8715
T2 classification	0/1	0.1177
T3 classification	0/1	0.6795
T4 classification	0/1	1.2836
N+ classification	0/1	0.3623
Tumor location is non-glottic	0/1	0.2644
Hemoglobin level	number^*^	−0.3190
Total radiation dose	number	−0.0034

* in mmol/l

The 0/1 input is the binary answer to the yes or no question of the model feature.

### Model validation and statistics

A Receiver Operating Characteristic (ROC) curve computation module was used to compare the predicted survival with the actual survival of each patient population [18]. For the training cohort we applied an internal validation on the entire dataset. We compared internal validation to the external validations on the clinical and trial cohort. All validations result in an area-under-the-curve (AUC) that displays how well the model predicted the survival of the patients. An AUC of 0.5 indicates that the result is completely random meaning that the model is not able to predict outcome and an AUC of 1.0 indicates that the result is perfectly matched meaning the model is a perfect outcome predictor. Bootstrapping was used to determine the uncertainty in the model's AUC reported by the program. Specifically, the AUC was determined a thousand times using the bootstrap function provided by the Matlab statistics toolbox. All cohorts were bootstrapped and in each bootstrap sample the model was applied to determine +/− 2 standard deviations of the AUC in all cohorts. Additionally, the predicted probability of survival was compared to the observed probability of survival for each prognosis group to assess the calibration of the model for each cohort.

### Reference cohorts

To compare the effectiveness of the model in the clinical cohort we used the trial cohort (the RTOG-91-11 trial dataset [7]) and the training cohort (MAASTRO dataset [18]). With respect to the randomized RTOG-91-11 trial, only the patients treated with radiation only were selected (*n* = 177). The training and trial cohort were added to separate databases with the same data structure as the clinical cohort, this enabled the use of the same software for analyzing each cohort separately. An overview of the patient population of these datasets is given in Table 2. To perform a univariate survival analysis, the Kaplan Meier method was used. The prognosis groups were divided into 3 groups, classifying the 25% lowest survival predictions and 25% highest survival predictions as the poor and good prognosis group respectively. The middle 50% were classified as medium prognosis. The training cohort was used to create these thresholds for the poor, medium and good prognosis groups. These survival prediction thresholds dividing the training cohort were also applied to the clinical and trial cohort model outcomes. To compare the Kaplan Meier curves between cohorts and between prognosis groups the log-rank test was used. In all statistical tests p-values of less than 0.05 were assumed to indicate statistical significance.

**Table 2:** Patient population model input parameter values

		Training Cohort		Clinical Cohort		Trial Cohort		Training VS	
		#	%	#	%	#	%	Clinical	Trial
Total		978		52		177			
Age	47-60 years	357	37	15	29	96	54	*p* > 0.20	*p* < 0.05
	> 60 years	621	63	37	71	81	46	*p* > 0.20	*p* < 0.05
Gender	Male	870	89	47	90	136	77	*p* > 0.20	*p* < 0.05
	Female	108	11	5	10	41	23	*p* > 0.20	*p* < 0.05
T-classification	T1	524	54	18	35	0	0	*p* < 0.05	*p* < 0.05
	T2	260	27	11	21	18	10	*p* > 0.20	*p* < 0.05
	T3	128	13	14	27	144	81	*p* < 0.05	*p* < 0.05
	T4	66	7	7	13	15	8	*p* = 0.07	*p* > 0.20
	Missing	0	0	2	4	0	0	*p* < 0.05	*p* > 0.20
N-classification	N0	884	90	41	79	92	52	*p* < 0.05	*p* < 0.05
	N+	98	10	11	21	85	48	*p* < 0.05	*p* < 0.05
	Missing	2	0	0	0	0	0	*p* > 0.20	*p* > 0.20
Tumor location	Glottic	723	74	27	52	49	28	*p* < 0.05	*p* < 0.05
	Non-Glottic	255	26	25	48	128	72	*p* < 0.05	*p* < 0.05
Hemoglobin level	Low^1^	168	17	24	46	58	33	*p* < 0.05	*p* < 0.05
	Normal-high	667	68	28	54	116	66	*p* < 0.05	*p* > 0.20
	Missing	0	0	0	0	3	2	*p* > 0.20	*p* < 0.05
Total radiation dose	<60Gy	16	2	11	21	5	3	*p* < 0.05	*p* > 0.20
	60-66Gy	437	45	22	42	1	1	*p* > 0.20	*p* < 0.05
	>66Gy	541	55	19	37	171	97	*p* < 0.05	*p* < 0.05

1 Male < 8.5 mmol/l, Female < 7.5 mmol/l)

The clinical and trial cohort are compared to the training cohort to indicate a statistical difference.

## RESULTS

The data mining of the OIS resulted in an initial clinical cohort of 125 patients primarily diagnosed with laryngeal carcinoma. From this cohort, patients with missing data were then excluded; 13 patients because the diagnosis was not older than 2 years and thus it is impossible to assess 2-year survival for these patients; 3 patients due to a lack of treatment dose available in the OIS; 57 patients (the largest exclusion group) due to a lack of hemoglobin measurements before treatment. This resulted in a clinical cohort containing 52 patients diagnosed between June 1993 and February 2012 with complete datasets suitable for analysis, at the time of modelling; in time the set will automatically grow.

**Figure 1:**
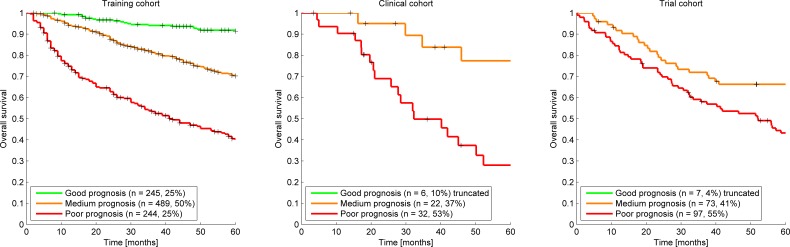
Kaplan Meier Curves for each cohort The survival prediction thresholds to create the poor, medium and good prognosis groups were 57% and 81% chance of 2-year survival. This resulted in a group distribution of 53%, 36% and 10% and 53%, 42% and 5% for the poor, medium and good prognosis group for the clinical cohort and trial cohort respectively, while (by definition) the training cohort had 25%, 50% and 25% distribution.

The model predicted prognostic survival groups, resulted in the Kaplan Meier curves presented in Figure 1. The survival prediction thresholds to seperate the poor, medium and good prognosis groups were 58% and 82% chance of 2-year survival. By definition these thresholds meant that the training cohort had 25%, 50% and 25% of patients in the poor, medium and good prognosis groups respectively. Applying the same thresholds to the clinical cohort gave a group distribution of 53%, 36% and 10% and for the trial cohort gave 55%, 41% and 4%. The Kaplan Meier curves of all groups in the clinical and trial cohort were compared with their equivalent in the training cohort. The survival prediction of each prognosis group in the clinical and trial cohort was not statistically different from the corresponding training cohort prognosis group (*p* > 0.2). The clinical cohort's poor and medium prognosis groups were statistically different (*p* < 0.05) but the medium and good prognosis groups were not (*p* > 0.2). The trial cohort comparison showed similar results.

**Figure 2:**
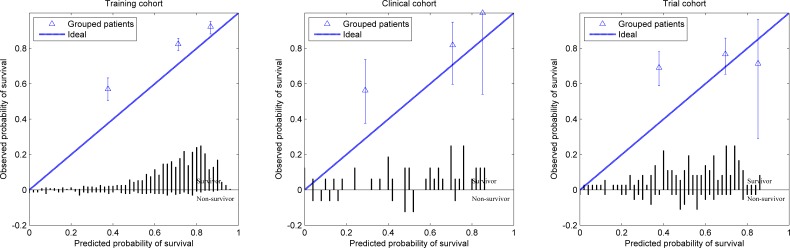
Calibration curve for each cohort showing the observed survival in relation to the predicted survival for the poor, medium and good prognosis groups The bar graph shows the survivor and non-survivor distribution per predicted survival probability bin.

The ROC computation resulted in AUC values of 0.77, 0.71 and 0.57 for training, clinical and trial cohort respectively. Bootstrapping (1000 samples) resulted in normally distributed AUC reliability intervals (+/−2SD) of 0.73 to 0.81, 0.55 to 0.88 and 0.47 to 0.67 for training, clinical and trial cohort respectively. The model calibration plots are presented in Figure 2. For the training cohort the observed 2-year survival is higher than predicted for the poor and medium prognosis group. The same can be concluded for the clinical cohort as the difference in survival for each prognosis group did not reach statistical significance (*p* > 0.2) between the training and clinical cohort. No statistical difference could be found between the prognosis groups of the trial cohort (*p* > 0.2) and the poor and good prognosis group survival was different from the training cohort (*p* < 0.05).

## DISCUSSION

We have implemented a survival prediction model for laryngeal carcinoma patients treated with primary radiation in a clinical cohort from a completely different geographical area (Australia *vs* The Netherlands) and evaluated the same model in a trial cohort from North America. In previous work this model was validated in other curated independent datasets from the Leuven Cancer Institute (Belgium), VU University Medical Center (Netherlands), Netherlands Cancer Institute-Antoni van Leeuwenhoek Hospital (Netherlands) and Christie Hospital (UK) [18]. The AUCs reported were 0.68, 0.74, 0.71 and 0.76 for each mentioned group respectively. The uncurated clinical cohort had a comparable accuracy (AUC of 0.71). The model is able to predict 2-year survival for laryngeal cancer patients for the clinical cohort as the AUC is statistically different from 0.5. The AUC reliability interval was larger than observed for the training cohort which can be explained by the smaller size of the clinical cohort. Comparable results were shown for the Leuven dataset (AUC 0.50 - 0.82) which is similar in size (*n* = 109). Another important fact is that the applied model was learned from the training cohort so it will by definition perform better on this cohort. The model calibration plots (Figure 2) shows that the model is not perfectly calibrated for 2-year survival, underestimating survival especially in the poor prognosis group. The likely reason is that this proportional hazard model was trained in the original study for 5-year survival prediction and provides a baseline survival for all time points between 0 and 5 years. It is not uncommon for these types of models to be recalibrated after acquiring more data to improve the survival probability [19]. The observed survival in the clinical cohort is not statistically different from the observed survival in the training cohort as reported earlier in the Results section demonstrating that the model performance in the clinical cohort is comparable to the training cohort. The prognosis distribution of the clinical cohort is shifted towards the poor prognosis group; the main reason is the difference in patient population. As shown in Table 2 the clinical cohort patients have more T3 and T4 cancers, more often have N1 and N2 disease, more non-glottic cancers, receive a lower treatment dose and have lower hemoglobin levels. These are all unfavorable predictors for survival in the prediction model. The more advanced cancers and nodal metastasis might be explained by the socioeconomic difference between the Illawarra and Maastricht regions, as patients are referred to the ICCC at a later stage or wait longer to consult their physician. However, the observed survival is not statistically different from the training cohort for each prognosis group as reported in the results. This indicates that the training and clinical cohort are similar and that similar features seem to be predictive for survival. The inclusion of the clinical cohort patients in a future training cohort may find additional features specific to the poorer prognosis group, which is the subject of future work.

Uncurated clinical data has been demonstrated to be sufficient to produce and validate useful models and DSS, however the work also indicates that prospective consistent data recording can improve opportunities to learn from clinical data. Increasing the numbers of patient records eligible to be entered into the modelling process can enable the addition of more model parameters and strengthen model performance. For this study the data quality was very high in comparison to similar studies [20] where less than 5 percent of the treated patient records were usable after data mining while in this study over 30 percent of the treated patient records have been included. This can be explained by a previous retrospective study in this patient group. The original data for these patients was complimented with great detail, something that is not standard in a radiotherapy clinic.

**Figure 3:**
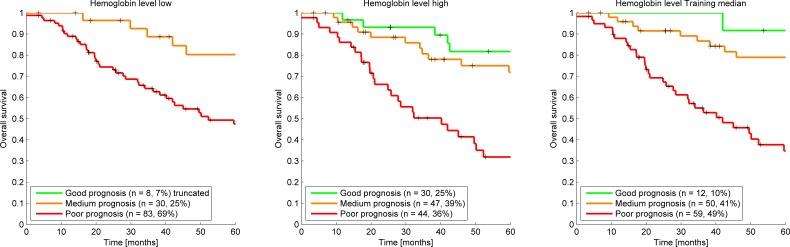
Kaplan Meier survival curves clinical cohort using a low, high, and training median imputation value demonstrating the effect of assuming hemoglobin values for the 57 patients in the clinical cohort that were missing a hemoglobin level measurement before treatment

The largest gap in data was caused by the poorly recorded hemoglobin level measurements before the start of radiotherapy. Because hemoglobin level is one of the input parameters with the strongest weight in the model a separate analysis was undertaken where the hemoglobin level was imputed with a low (7.0mmol/l), high (11.0mmol/l) and the training set median (9.1mmol/l) value. This imputation resulted in an enlarged clinical cohort of 109 patients. This resulted in an AUC interval increase of 0.60 to 0.83, 0.61 to 0.84, and 0.62 to 0.85 for the mentioned imputations respectively, intervals which are somewhat tighter than for the non-imputed clinical cohort which is likely caused by the increase in patient numbers. The different imputation methods for the hemoglobin level resulted in a very different distribution of the patients across survival groups as shown in the Kaplan Meier curves (Figure 3). This shows that the model is very sensitive to the hemoglobin level and that one has to take great care in choosing an appropriate imputation method but it also shows that the clinical cohort contains more information that could be utilized. In future work smarter ways of imputing missing values could be explored. An example of a smarter solution is a Bayesian Network model where for example the hemoglobin level could be derived by considering all other patient properties available instead a of simple median calculation [21].

In both the previous study [18] and in this study the model is able to predict survival for the clinical cohorts. However, in this study we show that the model is not able to predict 2-year survival for laryngeal cancer patients for the trial cohort: The AUC shows that the prediction is not different from random and the calibration plot shows that the observed survival is statistically the same for each prognosis group in the trial cohort. The comparison between the trial cohort prognosis groups as reported in the results confirms this. The most likely cause of this poor performance and calibration is that the trial patient population is different from the training and clinical cohort. Table 2 shows cohort properties which are statistically different between training and trial cohort. The trial cohort consists of younger patients, more females, almost exclusively T3 staged cancers, more nodal involvement, more non-glottic cancers and almost exclusively high radiation dosages. This patient-type is under represented in the training cohort. Although data quality is extremely high in a clinical trial, the patients in the trial cohort are highly selected being relatively young patients with advanced cancers that were treated perhaps with a higher quality level or with different treatments than standard practice or that were different by another unknown confounding factor. After training new model coefficients on the trial cohort and using the same data for validation the AUC was only 0.58. Even when using an optimistic overestimating validation, the model performs poorly, this means that the data does not contain the knowledge we need to predict survival for this specific cohort supporting our earlier statement that there might be another unknown factor that is of great influence on the survival for these patients. This difference between clinical routine and trial cohorts is one of the arguments against using solely evidence from clinical trials as the source of clinical guidelines in radiation oncology [22, 23]. For rapid learning, if a training cohort is different from a validation cohort and/or the patients in the validation cohort are underrepresented in the training cohort, a poor performance of the model can be expected. To increase model performance more patients with different characteristics should be included in the training set during model learning. Including trial patients can have a negative effect on model performance; it has been reported that it can result in a biased model towards this population. As an example, the predicted survival (i.e. the calibration of the model) will be much higher than can be obtained in routine clinical practice as routine quality is expected to be lower than in clinical trials, which is known to affect survival [24]. The poor performance in the trial cohort also underlines the need of model commissioning. Before using any prediction model, it is important to verify if this model does indeed perform well in a specific population. Commissioning of hardware and software is a well-known process in radiotherapy and decision aid tools should undergo the same quality assurance procedures. A better modelling approach would be multi center rapid learning systems that can enable the model learning algorithm to learn from data present in multiple centers, using different machine learning approaches, such as Bayesian Networks, which can explicitly account for biased datasets. This could include the integration of large scale observational studies such as DAHANCA [25] and the integration of clinical routine and clinical trial cohorts as suggested by others [13, 26]. In ongoing work on the rapid learning infrastructure, we use Semantic Web technology and ontologies such as the Radiation Oncology Ontology [https://bioportal.bioontology.org/ontologies/ROO] to create well defined semantically interoperable data stores and secure messaging systems to facilitate multi institutional rapid learning.

In clinical practice TNM staging is used to estimate the prognosis for larynx patients and there is some evidence of additional single variable prognosis predictors [27, 28]. To our knowledge there are no models to predict survival in laryngeal cancer patients. Some survival prediction models exist for other head and neck cancer patients [29, 30]. The study [30] with a similar approach, large training and external validation cohorts found comparable results in model performance.

## CONCLUSIONS

We were able to use a data mining system to automate the collection of model parameters in a totally different clinical cohort in a different country and healthcare system and predict 2-year survival for their patient population using uncurated clinical data. The study shows that routine clinical data contains valuable information that could be harvested to improve and personalize patient care and even more so if recorded in a detailed, structured manner. The results demonstrate that further investigations into the difference between clinical trial cohorts and clinical cohorts is necessary with the potential for rapid learning systems to provide evidence for patients who do not fit clinical trial criteria.
